# Histological evaluation of the effect of low-frequency electric stimulation on healing Achilles tendons in rats

**DOI:** 10.1590/ACB360103

**Published:** 2021-02-01

**Authors:** Sharbo Martins Casagrande, Maria de Lourdes Pessole Biondo-Simões, Sergio Ioshii, Rogério Ribeiro Robes, Rachel Biondo-Simões, Bruno Russiano de Oliveira Boeno

**Affiliations:** 1 Fellow Master degree. Universidade Federal do Paraná – Postgraduate Program in Clinical Surgery – Curitiba (PR), Brazil.; 2.Full Professor. Universidade Federal do Paraná – Department of Surgery; and Permanent Professor. UniversidadeFederal do Paraná – Postgraduate Program in Clinical Surgery – Curitiba (PR), Brazil.; 3 Full Professor. Universidade Federal do Paraná – Department of Pathology; and Pontifical Universidade Católica do Paraná – Curitiba (PR), Brazil.; 4 Physician. Veterinary Hospital; and Fellow Master degree. – Universidade Federal do Paraná – Postgraduate Program in Clinical Surgery – Curitiba (PR), Brazil.; 5 PhD. Oncology Service, Hospital Angelina Caron and Fellow Master degree – Universidade Federal do Paraná – Postgraduate Program in Clinical Surgery – Curitiba (PR), Brazil.; 6 Graduate student. Universidade Federal do Paraná – Curitiba (PR), Brazil.

**Keywords:** Electric Stimulation, Collagen, Achilles Tendon, Wound Healing, Rats

## Abstract

**Purpose:**

Histologically evaluate the effects of low frequency electrical stimulation in the treatment of Achilles tendon injuries in rats.

**Methods:**

Thirty-four rats underwent Achilles tendon tenotomy and tenorrhaphy. They were randomly allocated in two groups. Half of the sample constituted the experiment group, whose lesions were stimulated with 2 Hz, nonpolarized current and 1 mA, for 14 days. The other animals formed the control group. They were evaluated at 2, 4 and 6 weeks. The histological study was carried out, the collagen density and the wound maturity index were measured.

**Results:**

The healing score was higher in the group stimulated at the 6th week (p = 0.018). The density collagen 1 was higher in the group treated at the three times (p = 0.004) and that collagen 3 was higher in the group treated at 6 weeks (p = 0.004). Together, collagen 1 and 3 were higher in the group stimulated at 4 and 6 weeks (p = 0.009, p = 0.004). The maturity index was higher in this group at the three moments (p = 0.017 p = 0.004 and p = 0.009).

**Conclusion:**

Low frequency electric stimulation improved healing and increased the quantity of collagen.

## Introduction

Degenerative or traumatic tendon lesions are very frequent, and lesions in the Achilles tendon are among the most frequent, corresponding to 20 to 50% of all lesions and increasingly associated with practicing sports^1^-^3^.

Achilles tendons injuries present high levels of morbidity and complications. The consequence of poor-quality healing due to hypovascularization means that there is a high rate of recurring ruptures and complications, in addition to prolonged rehabilitation^1^,^3^.

There is no consensus regarding the best method of treatment. Some studies claim that there are no significant differences between surgical and conservative methods in the long term^4^,^5^. Some studies have shown that the recurrence rate of lesion after surgical treatment is significantly lower than that of non-surgical treatments^1^,^6^-^8^.

Complications, generally related to healing deficiencies, can occur in both the tendon and the skin. The most common are new ruptures, which may be early or late, dehiscences, infections and necrosis of the skin^1^,^2^,^6^,^7^,^9^.

Electric stimulation has shown controversial results. The outlook of some studies regarding its use is promising, as it improves cutaneous perfusion with better healing of tendons, skin, bones and ligaments^10^,^11^. For Folha *et al*.^12^, the results were inconclusive. A comparison of the diverse studies has become difficult due to a lack of parameterization^11^,^13^. Some studies conducted on animals have shown that electric stimulation improves osteogenesis in rabbit fibula and the epithelialization of surgical wounds in pigs^14^,^15^.

In a histopathological analysis, some studies have shown a lower quantity of granulation tissue and a higher quantity of aligned collagen bundles^16^,^17^, an increase in the concentration of adenosine triphosphate (ATP), amino acid uptake and protein synthesis in human and animal skin fibroblasts^18^,^19^.

Clinical studies have shown that the application of low-intensity microcurrents aided the healing of the area of skin ulcers, requiring a lower number of debridement and keeping wounds healed for longer. This condition was associated with lower infection rates^20^,^21^.

The search for therapies that facilitate the healing process of all types of tissue has been constantly addressed. The best solution for Achilles tendon lesions would mean fewer complications and early rehabilitation. Electric stimulation could be an option, as it is a simple and easily accessed therapy.

The aim of this work is to evaluate the influence oflow-frequency electric stimulation on the healing of Achilles tendons in rats after surgical repair.

## Methods

### Ethical evaluation

The project from which this study originated was analyzed and approved by the Ethics Committee on the Use of Animals at the Department of Biological Sciences of the Universidade Federal do Paraná (UFPR), with Approval Certificate 1170. The study complied with the norms of Federal Law 11.797 of 08 October 2008, regulated by Decree 6.899 of 15 July 2009.

### Sample, accommodation conditions and constitution of the groups

Rats were chosen as the biological model. Thirty-four male Wistar rats (*Rattus norvegicus albinus*) from the UFPR animal facility (vivarium) were used, aged 200 ± 5 days and weighing 517.4 ± 57.3 g.

The animals were kept in polypropylene boxes, suitable for the species, containing white shavings, with four animals per cage. They were provided with water and standard commercial feed suitable for the species *ad libitum*. The light-dark cycle was 12 hours, the room temperature was 20 ± 2 °C, and there was no artificial regulation of the relative humidity of the air.

The animals were randomly allocated to two groups. Group A was the control group and Group B was the experiment group. All were submitted to tenotomy and tenorrhaphy. Group B was given electric stimulation at 2 Hz. The animals were evaluated after 2, 4 and 6 weeks.

### Surgical procedure

The experiment was conducted at the Laboratory of the Discipline of Surgical Techniques and Experimental Surgery of the UFPR.

The anesthesia and analgesia were handled by the laboratory’s veterinary doctor. For the preanesthetic medication, an intramuscular injection of ketamine hydrochloride 50 mg/kg combined with xylazine hydrochloride 2 mg/kg was used. The anesthetic was induced through inhalation with 1% isoflurane and maintenance with the same drug at 1.5% under a mask with 100% oxygen^22^.

Following recovery from the anesthesia, the animals were given an intramuscular administration of sodium dipyrone monohydrate 50 mg/kg, maintained every 12 hours for analgesia^22^.

**Figure 1:**
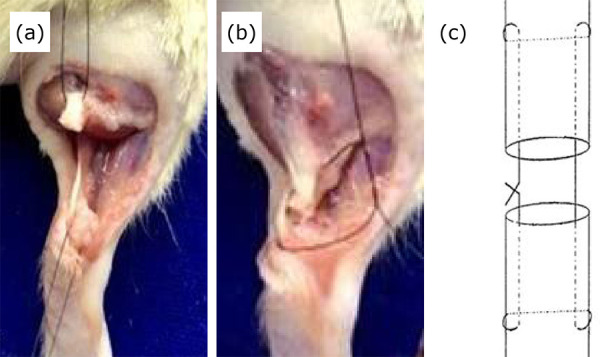
Surgical procedure. (a) Achilles tendon isolation and complete tenotomy, followed by (b and c) tenorrhaphy with modified Kessler stitch.

After they were weighed and identified, the posterior region of their foot was shaved using an electric device. The antisepsis procedure was performed using polyvinylpyrrolidone-iodine. The skin was then incised, the Achilles tendon was isolated and a complete Achilles tenotomy was performed, i.e., of the lateral and medial band. Tenorrhaphy was then performed with 6-0 nylon monofilament thread using modified Kessler stitches and skin synthesis with simple stitches with 4-0 nylon monofilament thread (Fig. 1).

For the animals in Group A, the procedure consisted of a tenotomy followed by tenorrhaphy, and for those in Group B, immediately after the surgical procedure, the first session of electric stimulation was added. The electric therapy was performed under analgesia^23^,^24^. The animals in this group were given 20 min of electric stimulation every day for 14 days.

### Electric stimulation

**Figure 2:**
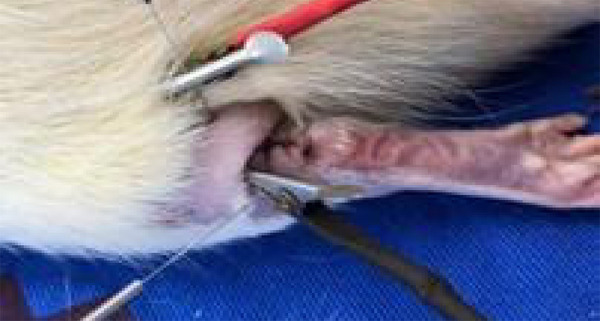
Insertion of 0.25 × 30 mm needle proximal and distal to the tenorrhaphy of the Achilles tendon for electrostimulation.

For the electric stimulation, the Nkl 608 digital device, serial number EM 4544, was used, approved by the National Health Surveillance Agency (ANVISA), reviewed and calibrated by the factory. An alternating current was used, i.e., without the formation of a positive and negative pole, a nonpolarized current, biphasic rectangular pulse of 600 μs, intensity of 1 mA, frequency of 2 Hz. Acupuncture needles (0.25 × 30 mm) were used 1 cm distal and proximal to the surgical site. For this, the needles were positioned 1.0 and 2.0 cm above the calcaneum joint. The electrodes were attached to the needles (Fig. 2).

### Euthanasia

Seven animals from each group were euthanized after 2, 4 and 6 weeks. The right tendons were resected for the histological study.

Euthanasia was performed under anesthesia induced by venipuncture of the caudal vein and administration of 15 mL sodium thiopental and after 10% potassium chloride. Euthanasia was induced with inhaled anesthetic after venipuncture of the caudal vein and 15 mg of sodium thiopental and 10% potassium chloride were administered.

### Histopathological evaluation

The gastrocnemius muscle and the Achilles tendon containing the scar area in question were removed from the bony protrusions and the samples, set in 10% formalin, were forwarded for histological processing. From the paraffin blocks, 4 mm thick cuts were removed and mounted on slides, which were subjected to staining using the techniques of hematoxylin and eosin and Sirius Red. The slides were evaluated by a pathologist blinded to the groups.

The cuts stained by hematoxylin and eosin were evaluated using an Olympus BX51 (Tokyo, Japan) common optical microscope. Twelve histopathological parameters were observed in accordance with Stoll^25^: organization of the extracellular matrix; myxoid material content; cellularity and matrix cell relationship; cell alignment; cell distribution; morphology of the nuclei; organization of repair tissue in the tendon callus; transition between the defect and normal tendon tissue; callus configuration; degenerative changes and tissue metaplasia; vascularization in the defect area; and inflammation, each with two to four variables. The variables of the twelve histopathological parameters of each sample were evaluated and a final arithmetic score, referred to here as the healing score, was obtained for statistical analysis.

The cuts with Picrosirius Red staining were examined under a polarized light using an Axio Scope.A1 microscope (Zeiss, Germany) with a coupled Axio Cam camera (Zeiss, Germany). The images were photographed and filed as standard JPEGs for later reading with Image-Pro Plus 4.5 software (Media Cybernetics, USA). In each cut, ten fields on the scar line were analyzed, with 100× magnification. In each one, the percentage of area occupied by red and yellow (collagen I) and green (collagen III) fibers was calculated. Considering that the other types of collagen constitute very small fractions, for practical purposes the sum of collagens I and III was considered as the total amount of collagen in the scar.

It was also possible to gauge the maturity index of the scars, obtained by the collagen 1/collagen 3 relationship.

### Statistical analysis

The results of the quantitative variables were described by mean, standard deviation, median, minimum value and maximum value. Categorical variables were described by frequency and percentage. To compare the 3 groups evaluated after 2, 4 and 6 weeks, with regard to the quantitative variables, the Kruskal-Wallis nonparametric test was used. At each evaluation, the control and experiment groups were compared using the Mann-Whitney nonparametric test. Fisher’s exact test was used for a comparative analysis of the categorical variables. Values of p < 0.05 indicated statistical significance. The data were analyzed using the computer program Stata/SE v.14.1. StataCorpLP, USA.

Initially, separate tests were conducted for the control and experiment groups for the null hypothesis that the results of the variable are the same for the three groups defined by each evaluation time (2, 4 and 6 weeks), versus the alternative hypothesis that the results are not all the same. In the case of a rejection of the null hypothesis, the evaluations were compared two by two. Following this, for each variable, at each of the evaluations (2, 4 and 6 weeks), the null hypothesis that the results of the control and experiment groups could be the same was tested, versus the alternative hypothesis of different results.

## Results

The value of an animal in the control group at 6 weeks, from analyzes related to collagen, was excluded due to errors in the collection of images that make it impossible to read.

### Healing score

**Figure 3:**
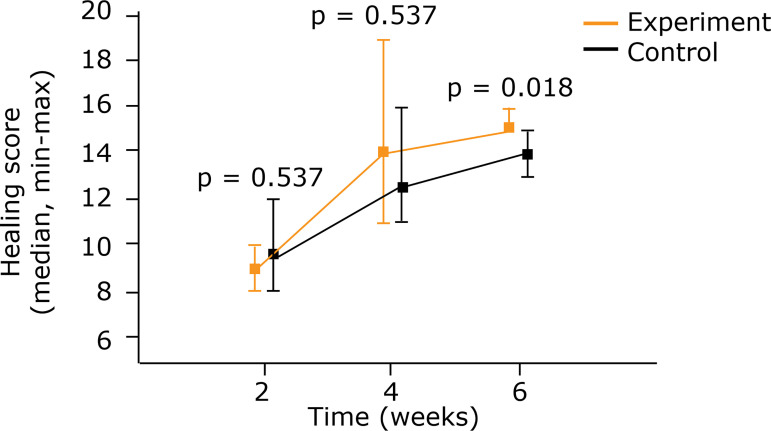
Comparison between control group and experiment for the healing score using medians and maximum and minimum values (Mann-Whitney nonparametric test).

An analysis of the healing score, in accordance with Stoll^25^, in an intragroup comparison, showed an improvement over time in both groups. A comparison between the control and experiment groups revealed better results in the experiment group after 6 weeks (p = 0.018) (Table 1) (Fig. 3).

**Table 1:** Intragroup comparative analysis of the healing score according to Stoll^23^

Group	Evaluation (weeks)	Healing Score													p*						
			n		Average		Median		Minimum		Maximum		Standard Deviation		2 × 4 × 6 weeks		2 × 4 weeks		2 × 6 weeks		4 × 6 weeks
A	2		6		9.80		9.50		8		12		1.5								
	4		6		13.2		12.5		11		16		1.9								
	6		7		14.1		14.0		13		15		0.7		0.004		0.003		< 0.001		0.194
B	2		5		9.20		9.0		8		10		0.8								
	4		5		14.4		14.0		11		19		3.0								
	6		5		15.4		15.0		15		16		0.5		0.008		0.002		< 0.001		0.146

* Kruskal–Wallis nonparametric test p < 0.05

** A = Control group; B = Experiment group.

### Collagen density

**Figure 4:**
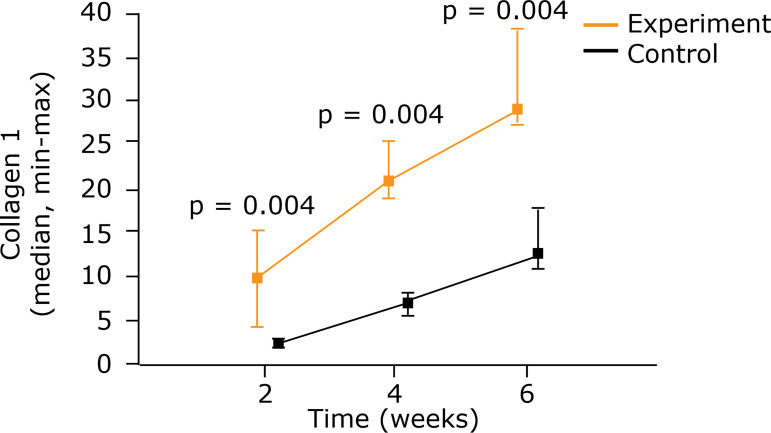
Comparison between control group and experiment for type 1 collagen using medians and maximum and minimum values (Mann-Whitney nonparametric test).

The type 1 collagen, in an intragroup comparison, showed a gain at the three times in question. A comparison of the two groups showed greater density at all three times in the group that had been given electric stimulation (p = 0.004) (Fig. 4) (Table 2).

**Table 2:** Intragroup comparative analysis of type 1 collagen.

Group		Evaluation (weeks)	Type 1 collagen													p*						
				n		Average		Median		Minimum		Maximum		Standard Deviation		2 × 4 × 6 weeks		2 × 4 weeks		2 × 6 weeks		4 × 6 weeks
A		2		6		2.31		2.31		1.92		2.73		0.31								
		4		6		6.80		6.98		5.43		8.07		0.96								
		6		6		13.1		12.6		10.8		17.7		2.52		0.001		< 0.001		< 0.001		< 0.001
B		2		5		10.1		9.8		4.07		15.3		4.14								
		4		5		21.7		20.8		18.9		25.5		3.08								
		6		5		30.8		29.0		27.2		38.1		4.34		0.002		0.001		< 0.001		< 0.001

* Kruskal–Wallis nonparametric test p < 0.05

** A = Control group; B = Experiment group.

**Figure 5:**
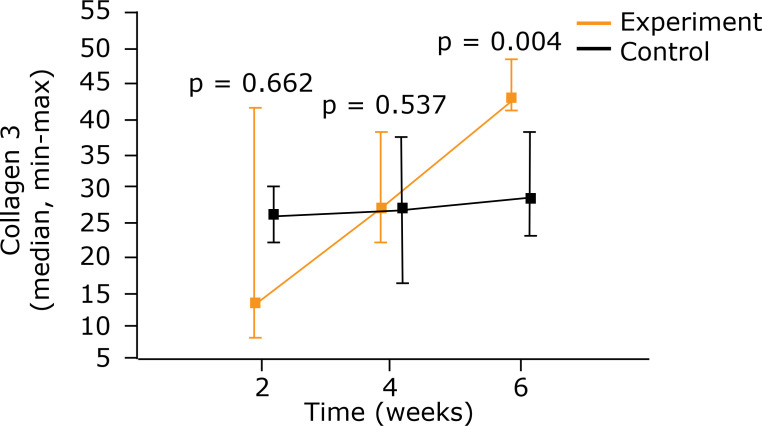
Comparison between control group and experiment for type 3 collagen using medians and maximum and minimum values (Mann–Whitney nonparametric test)

When an intragroup analysis of the type 3 collagen was performed, increased density was observed over time in both the control and experiment group. However, the differences between the three evaluations in the control group were not significant (p = 0.581). In the experiment group, the density increased as the process evolved (p = 0.011). A comparison of the groups, nevertheless, only showed a significant difference in the sixth week when higher density was found in the experiment group (p = 0.004) (Fig. 5) (Table 3).

**Table 3:** Intragroup comparative analysis of type 3 collagen.

Group		Evaluation (weeks)	Type 3 collagen													p*						
				n		Average		Median		Minimum		Maximum		Standard Deviation		2 × 4 × 6 weeks		2 × 4 weeks		2 × 6 weeks		4 × 6 weeks
A		2		6		26.4		26.1		22.1		30.3		3.39								
		4		6		27.3		27.0		16.4		37.5		7.82								
		6		6		30.3		28.7		22.9		38.1		6.20		0.581						
B		2		5		21.6		13.4		8.25		41.8		15.0								
		4		5		30.3		27.3		22.2		38.4		7.42								
		6		5		44.9		43.4		41.2		48.8		3.47		0.011		0.400		0.001		0.004

* Kruskal–Wallis nonparametric test p < 0.05

** A = Control group; B = Experiment group.

**Figure 6:**
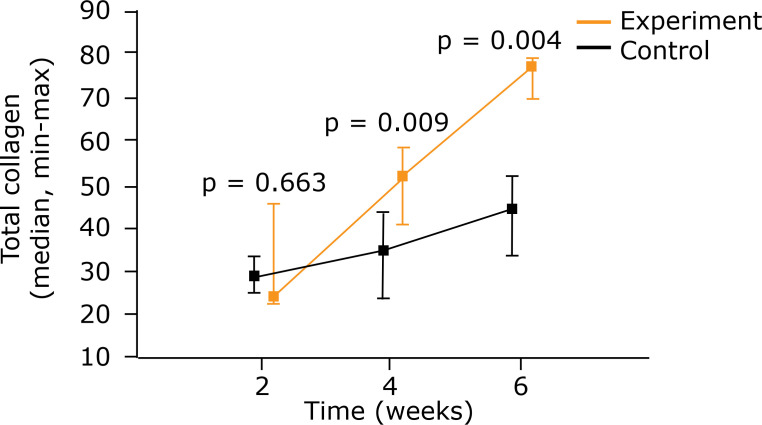
Comparison between control group and experiment for the amount of total collagen using medians and maximum and minimum values (Mann-Whitney nonparametric test).

Considering the sum of the two types of collagen, it was perceived that in both groups the quantity increased over time. A comparison of the groups showed more collagen in the experiment group after 4 weeks (p = 0.009) and 6 weeks (p = 0.004) (Fig. 6) (Table 4).

**Table 4:** Intragroup comparative analysis of total collagen.

Group		Evaluation (weeks)	Total Collagen													p*						
				n		Average		Median		Minimum		Maximum		Standard Deviation		2 × 4 × 6 weeks		2 × 4 weeks		2 × 6 weeks		4 × 6 weeks
A		2		6		28.7		28.6		24.6		32.7		3.34								
		4		6		34.1		34.7		23.7		43.6		7.28								
		6		6		43.4		44.2		33.7		52.0		7.24		0.010		0.080		0.001		0.034
B		2		5		31.7		23.6		22.5		45.9		11.8								
		4		5		52.0		51.7		41.1		58.8		6.99								
		6		5		75.6		77.1		69.9		79.3		3.63		0.003		0.006		<0.001		0.003

* Kruskal–Wallis nonparametric test p < 0.05

** A = Control group; B = Experiment group.

**Figure 7:**
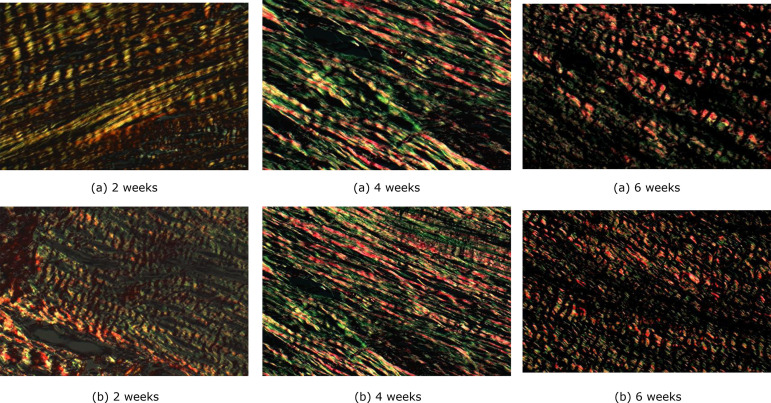
Photomicrographs of histological sections, from the three periods studied (Picro-Sirius Red, under polarized light - 100×) Green = collagen 3. Red = collagen 1. (**a**) Control group; (**b**) Experiment group.

**Figure 8:**
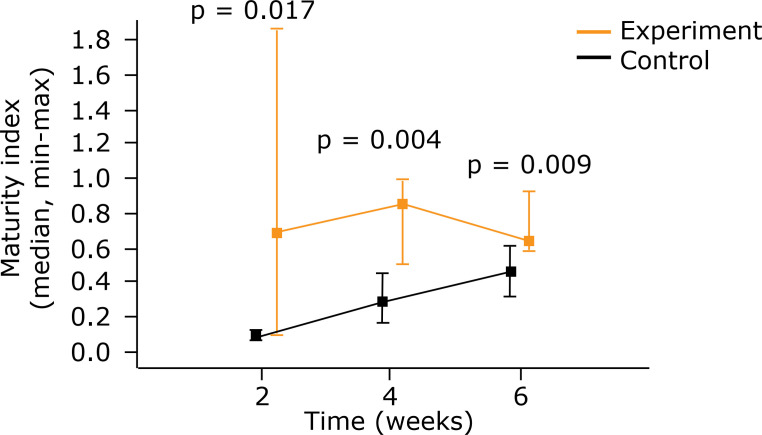
Comparison between control group and experiment regarding maturity index using medians and maximum and minimum values (Mann-Whitney nonparametric test).

The maturity index was higher in the experiment group at the three times in question (at 2 weeks p = 0.017, at 4 weeks p = 0.004 and at 6 weeks p = 0.009) (Table 5) (Figs. 7 and 8).

**Table 5:** Maturity index (collagen 1/collagen 3 ratio).

Group		Evaluation (weeks)		Maturity index												p*						
				n		Average		Median		Minimum		Maximum		Standard Deviation		2 × 4 × 6 weeks		2 × 4 weeks		2 × 6 weeks		4 × 6 weeks
A		2		6		0.089		0.087		0.064		0.115		0.018								
		4		6		0.274		0.284		0.159		0.445		0.107								
		6		6		0.444		0.455		0.319		0.600		0.098		0.001		< 0.001		< 0.001		0.007
B		2		5		0.802		0.682		0.097		1.855		0.700								
		4		5		0.756		0.850		0.494		0.993		0.221								
		6		5		0.692		0.639		0.580		0.925		0.140		0,990		-		-		-

* Kruskal–Wallis nonparametric test p < 0.05

** A = Control group; B = Experiment group.

## Discussion

Among the tendon injuries of the lower limbs, one of the most frequent is the rupture of the Achilles tendon. Considering that this tendon connects the calf muscles to the calcaneus, its function in propelling the foot when walking is understood, as is the importance of achieving good rehabilitation. It has been found that the rupture of this tendon affects individuals between the ages of 25 and 40. In this group, it is often associated with the practice of sports, with high-energy injuries, and there is a new peak of occurrence in individuals over the age of 60. In this latter group, ruptures are generally spontaneous and related to degeneration^26^.

The intention behind the treatment of these lesions is that the patient will recover the original strength of the tendon, but for this to occur it is necessary to make a full recovery. There is no consensus regarding the best option for treatment. It is possible to institute conservative treatment with early functional rehabilitation or surgical treatment^27^. There are several forms of treatment for acute ruptures that show good results, as long as they are associated with the early rehabilitation protocol^28^.

Opting for surgical treatment may lead to complications, the most frequent of which is related to the healing of the tendon or the skin due to poor local vascularization^1^,^2^,^6^-^8^.

Although it is not entirely clear how the mechanisms of electric stimulation affect the healing process and more studies are required, it seems that this supportive method of treatment has been shown to be safe and effective, improving the vascularization of the dermis, bones, tendons and ligaments^11^.

Nessler *et al*.^17^ electrically stimulated the tendons of rabbits *in vitro* and found that after 7 days they incorporated 91% more proline than the control group (p = 0.0272). The activity was also greater in the stimulated group for 42 days. According to Cheng *et al*.^29^, electric stimulation can guide the organization and morphology of the collagen obtained from tendons. Wang *et al*.^30^ demonstrated that electric stimulation could increase the migration of mesenchymal stem cells in rats.

Human fibroblasts electrically stimulated *in vitro* with a galvanic current and high voltage showed a significant increase in protein and DNA synthesis^20^. In a literature review, it was found that transcutaneous electric stimulation can improve the healing of wounds, the repair of tendons and the feasibility of skin flaps. The authors attempted to explain this by saying that there is greater production of the P substance and the peptide genetically related to calcitonin, which increase the local blood flow, facilitating the healing process^31^.

A study conducted in rats showed that electroacupuncture improved the connectivity between the anterior hypothalamus and the amygdala, leading to a greater formation of mesenchymal stem cells in the circulation. In this study, the authors reported an increase in serum IL-10. This would reduce the inflammatory response, creating an environment favorable to the differentiation of regulatory M2 macrophages. Thus, there would be better regeneration and remodeling^24^.

An experimental study involving rats evaluated electric stimulation at 2, 15 and 120 Hz in the inflammatory process and the expression of peripheral and central Cox2. It found that electric stimulation was effective in reducing edema in the inflammatory process. These results were clearer with 2Hz^32^.

Almeida *et al*.^33^,^34^ investigated the effect of electroacupuncture on the composition and organization of the extracellular matrix of the Achilles tendon of rats after a partial transection during the proliferative phase of healing. The results showed that there was no change in the concentration of noncollagen proteins or glycosaminoglycans or in the enzymatic activity of metalloproteinase-2 in the sectioned tendons. However, the hydroxyproline concentration increased significantly when these tendons were electrically stimulated. The birefringence analysis showed greater organization of collagen fibers in the treated group, and there was an increase in collagen concentration and better molecular organization of collagen fibers. When examined under an electron microscope, these fibers were thicker and more organized. According to the authors, this could improve the mechanical strength of the tendon following injury.

Folha *et al*.^12^ reported that high frequencies could have a negative effect on healing. When they used 100 Hz on rats’ tendons, they found less collagen formation and a poorer alignment of the fibers. A controversy arises here, given that Rampazo *et al*.^35^ used high-frequency electric stimulation at 120 Hz and high voltage comparing alternating currents (cathodic and anodic) and found no difference between the groups in the formation and alignment of collagen and in angiogenesis.

Alvarez *et al*.^15^ studied the healing of skin wounds in pigs and reported a highly significant increase in collagen synthesis from the fifth day onwards (p = 0.001) when submitted to electric stimulation. They also reported accelerated reepithelization. They suggested that electric stimulation could affect the proliferative and migratory capacity of epithelial cells and conjunctive tissue.

Jeon *et al*.^36^ observed that the use of a high-voltage pulsed current led to improved contraction and promoted healing in the skin wounds of rats by increasing the expression of transforming growth factor β1 (TGF-β1) and type 1 collagen synthesis.

Cheng *et al*.^19^ observed that electric stimulation increased ATP concentrations in the tissue and stimulated amino acid uptake into rat skin proteins. This contributed to the final increase in protein synthesizing. To these authors, the greater production of ATP could be explained by the movement of the protons, while the transport functions were controlled by the modification of the electric gradients through the membranes.

Some researchers have evaluated the realignment of collagen fibers using birefringence measures in the Achilles tendons. They found significant results with the application of pulsed ultrasound after a tenotomy by direct trauma^37^,^38^. However, Rampazo *et al*.^35^, using a pulsed current, did not find better collagen realignment or an increase in collagen 1 fibers when they treated Achilles tendon wounds in rats. A comparison of studies on electric stimulation is generally not possible most of the time because of the diversity of methodologies that are employed.

The application of 50 Hz, without informing the type of current used, revealed a greater quantity of cells, growth factors and greater maximum tension for the rupture of Achilles tendons in rats^39^. This same nonpolarized current frequency in rats’ Achilles tendons enabled the observation of an increase in angiogenesis and a higher number of fibroblasts with greater collagen density in the early phases of healing^40^.

Electric stimulation at a frequency of 10 Hz, without details of the current that was employed, showed greater maximum tension in rats’ tendons. This study did not take the transversal section of the tendon into account^41^. However, a study of rats that were electrically stimulated at a low frequency (10 Hz) and with a positive (anodic) polarized current confirmed the increase in the maximum tension of the tendons^42^. The use of 10 Hz with a high-voltage polarized current led to greater resistance in the group submitted to an anodic current than in the control group with a cathodic current^43^.

The use of an anodic or cathodic current is another contradictory point. Ahmed *et al*.^16^ studied the use of a 10-Hz polarized current in rabbits and found better healing and resistance with a cathodic current in the third week and with an anodic current in the fifth and eighth weeks.

A polarized current increases the risk of electrolysis and tissue lesions. For this reason, in the present study, a nonpolarized current was used as protocol. The parametrization most frequently used to treat musculoskeletal and neuropathic pains with low frequency (2-10 Hz) and nonpolarized currents, with the production of various neurotransmitters such as beta-endorphins, serotonin and norepinephrine^23^,^24^.

The results of the present study demonstrated that the healing process of the tendon with electric stimulation evaluated by the healing score, considering the 12 parameters of the scale of Stoll^25^ (which analyzed the organization of the extracellular matrix; myxoid material content; cellularity and matrix cell relationship; cell alignment; cell distribution; morphology of the nuclei; organization of repair tissue in the tendon callus; transition between the defect and normal tendon tissue; callus configuration; degenerative changes and tissue metaplasia; vascularization in the defect area; and inflammation) showed a similar evolution in both groups in the evaluations that were conducted after 2 and 4 weeks. A significant difference was only noticed after 6 weeks (p = 0.018) in favor of the treated group. It should be observed that this score was arrived at by the sum of each of the data that were examined. However, an isolated analysis of the healing score variables did not show a significant difference. An improvement in the healing score was seen after 4 weeks, comparing the control and experiment groups. However, no significant difference was shown, probably due to the small sample and the variability in the maximum and minimum values.

In this study, type 1 collagen showed a significant improvement at all times when electric stimulation was used (p = 0.004). Type 3 collagen, in the first two periods, was similar in the two groups in question. After 6 weeks, the group that had undergone electric stimulation had a higher amount of this type of collagen. If we consider that this collagen is more fibrillar and the one that appears earlier in regenerating tissues, we can assume that the stimulus for synthesis was prolonged by electric stimulation. The sum of the two types of collagen was shown to be greater in the electrically stimulated group in the evaluations that took place after 4 and 6 weeks, which leads us to reinforce the idea that the stimulation to synthesis was maintained for a longer time in this group.

Furthermore, an analysis of the maturity index obtained through the relationship of collagen 1 with collagen 3 showed that the scars of the group that underwent electric stimulation were more mature. This condition could be important with regard to improving the resistance of tendons.

It may be possible to explain these results through the improved vascularization^12^, the increased capacity of the conjunctive tissue cells to migrate to the area of the wound^15^, the increase in amino acid uptake^19^ and hydroxyproline synthesis^33^,^34^, the greater concentration of growth factors^39^, and the increased capacity for collagen synthesis by the fibroblasts^18^. The information gained through research has yet to provide the answer to all the questions and therefore much remains to be studied.

## Conclusion

Electric stimulation with a low-frequency (2 Hz) nonpolarized current improved the healing of rats’ Achilles tendons, led to better collagen synthesis and resulted in better scar maturity.
